# Human Properdin Released By Infiltrating Neutrophils Can Modulate Influenza A Virus Infection

**DOI:** 10.3389/fimmu.2021.747654

**Published:** 2021-12-09

**Authors:** Praveen M. Varghese, Shuvechha Mukherjee, Futwan A. Al-Mohanna, Souad M. Saleh, Fahad N. Almajhdi, Nazar Beirag, Saad H. Alkahtani, Reena Rajkumari, Beatrice Nal Rogier, Robert B. Sim, Susan Idicula-Thomas, Taruna Madan, Valarmathy Murugaiah, Uday Kishore

**Affiliations:** ^1^ Biosciences, College of Health, Medicine and Life Sciences, Brunel University London, Uxbridge, United Kingdom; ^2^ School of Biosciences and Technology, Vellore Institute of Technology, Vellore, India; ^3^ Biomedical Informatics Centre, Indian Council of Medical Research (ICMR)-National Institute for Research in Reproductive Health, Mumbai, India; ^4^ Department of Cell Biology, King Faisal Specialist Hospital and Research Centre, Riyadh, Saudi Arabia; ^5^ Department of Botany and Microbiology, College of Science, King Saud University, Riyadh, Saudi Arabia; ^6^ Department of Zoology, College of Sciences, King Saud University, Riyadh, Saudi Arabia; ^7^ INSERM U1104 Centre d’immunologie de Marseille-Luminy (CIML), Marseille, France; ^8^ Department of Biochemistry, University of Oxford, Oxford, United Kingdom; ^9^ Department of Innate Immunity, Indian Council of Medical Research (ICMR)-National Institute for Research in Reproductive Health, Mumbai, India

**Keywords:** innate immune system, complement system, complement evasion, human properdin, RNA viruses, influenza A virus, cytokine storm

## Abstract

The complement system is designed to recognise and eliminate invading pathogens *via* activation of classical, alternative and lectin pathways. Human properdin stabilises the alternative pathway C3 convertase, resulting in an amplification loop that leads to the formation of C5 convertase, thereby acting as a positive regulator of the alternative pathway. It has been noted that human properdin on its own can operate as a pattern recognition receptor and exert immune functions outside its involvement in complement activation. Properdin can bind directly to microbial targets *via* DNA, sulfatides and glycosaminoglycans, apoptotic cells, nanoparticles, and well-known viral virulence factors. This study was aimed at investigating the complement-independent role of properdin against Influenza A virus infection. As one of the first immune cells to arrive at the site of IAV infection, we show here that IAV challenged neutrophils released properdin in a time-dependent manner. Properdin was found to directly interact with haemagglutinin, neuraminidase and matrix 1 protein Influenza A virus proteins in ELISA and western blot. Furthermore, modelling studies revealed that properdin could bind HA and NA of the H1N1 subtype with higher affinity compared to that of H3N2 due to the presence of an HA cleavage site in H1N1. In an infection assay using A549 cells, properdin suppressed viral replication in pH1N1 subtype while promoting replication of H3N2 subtype, as revealed by qPCR analysis of M1 transcripts. Properdin treatment triggered an anti-inflammatory response in H1N1-challenged A549 cells and a pro-inflammatory response in H3N2-infected cells, as evident from differential mRNA expression of TNF-α, NF-κB, IFN-α, IFN-β, IL-6, IL-12 and RANTES. Properdin treatment also reduced luciferase reporter activity in MDCK cells transduced with H1N1 pseudotyped lentiviral particles; however, it was increased in the case of pseudotyped H3N2 particles. Collectively, we conclude that infiltrating neutrophils at the site of IAV infection can release properdin, which then acts as an entry inhibitor for pandemic H1N1 subtype while suppressing viral replication and inducing an anti-inflammatory response. H3N2 subtype can escape this immune restriction due to altered haemagglutinin and neuraminindase, leading to enhanced viral entry, replication and pro-inflammatory response. Thus, depending on the subtype, properdin can either limit or aggravate IAV infection in the host.

## Introduction

The complement system plays a crucial part in innate immunity and consists of cell-surface receptors and soluble factors that can recognise invading pathogens (1). Viruses can trigger the complement system, possibly through all three distinct target recognition pathways, i.e. classical, alternative and lectin pathways (2). Various soluble and membrane-associated proteins can regulate complement activation resulting in several effector functions contributing to the inactivation and elimination of viruses (3). These functions include viral opsonisation by complement components (4) promoting virolysis by membrane attack complex (MAC), enhanced phagocytosis, and modulating immune responses through the production of chemotactic factors and anaphylatoxins (5).

Human properdin acts as an up-regulator of the alternative pathway (6). The spontaneous hydrolysis of C3 continuously activates it into C3(H_2_O). C3(H_2_O) binds with factor B, a serine protease, forming a complex called C3(H_2_O)B. C3(H_2_O)B enables factor D to cleave factor B to Bb, yielding C3(H_2_O)Bb. This newly formed C3(H_2_O)Bb cleaves C3 to form C3b and C3a. The C3b binds to the pathogen surface as well as more factor B. These are then cleaved by factor D to create the C3 convertase C3bBb. C3bBb promotes the cleavage of C3 into C3a and C3b. C3b then binds with C3bBb to form the alternative pathway C5 convertase, C3bBbC3b. Properdin plays a critical role in stabilising the convertases by forming C3bBbP and C3bBbC3bP (7, 8). The stabilisation of the convertases helps improve their half-life, thereby enhancing the activity of the alternative pathway (9). C5 is cleaved by the C5 convertases to form C5a and C5b. The C5b produced acts as a nucleus for the MAC assembly (10), which is formed by the interactions of C6-C9. Finally, the MAC (C5b-9 complex) is inserted into the microbial surface, causing the lysis of the microbe (10).

In human serum, properdin circulates as cyclic polymers formed by head-to-tail association in the form of cyclic dimers, trimers and tetramers (11, 12), with plasma concentration of 22-25 µg/ml (13). The monomeric properdin (~53 kDa) is composed of non-identical seven thrombospondin type 1 repeats (TSRs) (14, 15). Each TSR molecule (TSR_1_-TSR_6_) contains six conserved cysteine residues, while the N-terminal domain, TSR_0_, is typically truncated (14, 15). TSR_4_ and TSR_5_ modules are considered crucial for C3bBb binding, suggesting the importance of both TSRs in stabilising the C3 convertase complex (12, 16–19). Properdin binding to microbial surfaces can lead to recruitment of fluid phase C3b, resulting in the assembly of C3bBb, further C3b deposition on the pathogen surface (20, 21), C5 convertase production, MAC formation, and then cell lysis. A recombinant form of TSR_4+5_, expressed as a double domain, has been shown to bind C3b, and inhibit the alternative pathway *in vitro* (22).

Previously, properdin has been reported to be involved in viral infection. Human umbilical vein endothelial cells (HUVEC), when treated with the extracellular vesicles from Kaposi’s sarcoma-associated herpesvirus (KSHV), were found to express properdin on their cell surface (23). Properdin was also found to bind HIV-1 glycoproteins, gp120 and gp41 (24), and thus, restrict HIV-1 interaction with the CD4 receptor and subsequent fusion of viral envelope with the cell membrane (24). Expression of properdin was also evident in CD8^+^ T cells, which were shown to play a critical role in viral elimination and clearance during infections (25, 26).

Influenza A Virus (IAV) causes a severe respiratory infection; the virus is classified into several subtypes, depending on the combination of their surface glycoproteins, hemagglutinin (HA) and neuraminidase (NA) (27). Although seasonal epidemics characterise IAV infection, sporadic and unpredictable global pandemic outbreaks occur due to antigenic variations within HA and NA glycoproteins (28). HA is a crucial glycoprotein required to bind sialic acid on the host cells, initiating cellular fusion and entry of virions (29). Different epitopes of HA can induce the synthesis of neutralising antibodies by B cells, permitting IAV particles to escape from immune defence mechanisms, resulting in seasonal epidemics (30). Cleavage of sialic acid moieties is mediated by NA, which enables the release of viral particles, thus, promoting dispersion of IAV (31).

This study was primarily aimed at investigating the complement-independent role of human properdin against IAV infection, using the H1N1 and H3N2 subtypes. Since neutrophils are known to secrete properdin from their specific granules, the ability of IAV to trigger a local release of properdin in the lung microenvironment was evaluated. The interaction of human properdin with IAV glycoproteins was established, followed by an assessment of the effect of properdin on IAV entry and replication, as well as the cytokine profile of lung epithelial cells (A549) infected with IAV subtypes. In this study, we report that properdin, on its own, can either restrict H1N1 or aggravate H3N2 IAV infection in the host cells.

## Material and Methods

### Viruses and Reagents

Influenza A Virus (IAV) subtypes, A/England/195/2009 (pH1N1) and the A/HK/1174/99 (H3N2), were obtained as described recently (32, 33). H1N1 and H3N2 pseudotyped viral particles were produced using the plasmids obtained from Addgene (32, 33). pcDNA3.1-swineH1-flag (H1 from swine H1N1 A/California/04/09) (Invitrogen), pcDNA3.1-swine N1-flag (N1 from swine H1N1 A/California/04/09), and pcDNA H3 (from A/Denmark/70/03 (H3N2) (Invitrogen) were purchased commercially. Plasmid pI.18-N2 [N2 from A/Texas/50/2012/(H3N2)] was provided by Dr Nigel Temperton (University of Kent). Anti-influenza viral antibodies and recombinant viral proteins were obtained from BEI Resources, National Institutes of Health, USA (32, 33).

### Purification of IAV Subtypes and Viral Titre Determination

The IAV subtypes investigated in this study were produced as previously described (32). Briefly, Madin-Darby Canine Kidney Cells (MDCK) were cultured in growth medium (DMEM-F/12 with GlutaMAX™ Supplement (Gibco) + 10% v/v Heat Inactivated Fetal Bovine Serum (FBS) (Gibco) + 1% v/v Penicillin-Streptomycin (PS) (Gibco) to 70% confluence in a 175 cm^2^ flask (Fisher Scientific). The cells were washed with Phosphate-buffered saline (PBS) (0.01M sodium phosphate, 0.0027M KCl, and 0.137M NaCl, pH 7.4 at 25°C) (Fisher Bioreagents) to remove any residual growth media. The cells were then challenged with H1N1 and H3N2 IAV subtypes diluted in serum-free DMEM (Gibco) for 1h at 37°C. Unbound virions were washed away with PBS and the cells were then incubated for 3 days at 37°C in infection medium [DMEM + 1% penicillin/streptomycin + 0.3% w/v bovine serum albumin (BSA) + 1 µg/ml of l-1-Tosylamide-2-phenylethyl chloromethyl ketone (TPCK)-Trypsin] (Sigma-Aldrich). Post incubation, the viral particles were harvested from the supernatant after centrifugation at 12,000 × g at 4°C for 45 min. The titre of the virus isolated was determined to be 1 × 10^6^ PFU/ml based on Tissue culture Infective Dose (TCID_50_) Assay using the Reed–Muench method (31).

### Production of Pseudotyped Lentiviral Particles

Pseudotyped lentiviral particles used were produced as previously described and characterised by western blotting (32). Briefly, HEK 293T cells were cultured in growth media to 70% confluence at 37°C under 5% v/v CO_2_. Cells were co-transfected using 20 µg of plasmids described in **Table 1**. The media was centrifuged at 5,000 × g for 10 min at 4°C to remove cell debris and the pseudotyped particles were harvested. The pseudotyped particles were concentrated by ultracentrifugation at 25,000 × g for 3 h at 4°C (33) and stored at -80°C until further use.

**Table 1:** Plasmids used for pseudotyped lentivirus particle production (33).

	H1N1	H3N2	VSV-G
Envelope Protein-Coding Plasmid	pcDNA3.1-swineH1-flag (H1 from swine H1N1 A/California/04/09) (Codon optimized H1 (Genecust))	pcDNA-H3 [H3 from A/Denmark/70/03/(H3N2)] (Codon optimized H3 (Geneart))	pCMV-VSV-G (Addgene plasmid # 8454)
	pcDNA3.1-swine N1-flag (N1 from swine H1N1 A/California/04/09) (Codon optimised N1 (Genecust))	pI.18-N2 (N2 from human H3N2 A/Texas/50/2012)	
Backbone Plasmid	pHIV-Luciferase (Addgene plasmid # 21375)		
Packaging Plasmid	psPAX2 (Addgene plasmid # 12260)		

### Purification of Native Human Properdin

Human native properdin was purified as previously described (22, 34). Briefly, IgG-depleted human serum (using Protein G-Agarose) was passed through a column containing CNBr-activated Sepharose (GE Healthcare, UK), coupled to a monoclonal antibody against human properdin (specific to TSR3). The column was washed with 3-bed volumes of HEPES buffer (10 mM HEPES, 140 mM NaCl, 0.5 mM EDTA, and pH 7.4), and the matrix-bound properdin was eluted using 3 M MgCl_2_. The peak fractions were dialysed against HEPES buffer overnight at 4°C. Minor contaminants were removed by passing through the HiTrap Q FF-Sepharose ion exchange column (GE Healthcare, UK).

### Isolation of Human Neutrophils and IAV Challenge

Human peripheral blood neutrophils were prepared by dextran sedimentation using heparinised whole blood obtained from healthy donors, in accordance with King Faisal Specialist Hospital and Research Centre Research Advisory Council (IRP) regulations using Ficoll-Paque (35). Contaminating RBCs were removed by hypotonic lysis with isotonic NH_4_Cl. The remaining cells were suspended in Krebs-HEPES medium (pH 7.4) containing 120mM NaCl, 1mM CaCl_2_, 1.2mM MgSO_4_, 4.8mM KCl, 1.2mM KH_2_PO_4_, 25mM HEPES, and 0.1% BSA. The neutrophils were purified through neutrophil isolation medium (Cardinal Associates, Santa Fe, USA) to yield 98% purity and 99% viability, as measured by flow cytometry and trypan blue exclusion tests. Isolated cells were suspended in Krebs/HEPES buffer at 5 × 10^6^ cells/ml and 0.1% BSA, pH 7.4.

Neutrophils were stimulated with H1N1 and incubated at 37°C overnight with gentle shaking. IL-6 (10 ng/ml) and the chemotactic peptide N-Formylmethionine-leucyl-phenylalanine (fMLP) (1µM) were used as positive controls. The neutrophils were centrifuged for 5 min at 10,000 x g, and supernatant was collected and concentrated using 10KD cutoff Amicon^®^ ultra-4 centrifugal filter units (UFC805024, Millipore). Samples were spiked with a constant amount of actin (10µg/ml), which were used as gel loading controls. Secreted properdin was detected by Western Blotting using rabbit anti-human properdin (1.19 mg/ml) (14); actin was detected by anti-actin antibody (mAb 4970, Cell Signaling Technology, MA, United States). ECL was performed using standard Horseradish peroxidase (HRP) labelled secondary antibodies.

### Direct Binding ELISA and Far-Western Blotting

ELISA and far-western blotting analysis were performed to determine the direct interaction of IAV subtypes with human properdin (32). Briefly, ELISA was conducted by coating constant concentration (20 µl) of whole viral particles (H1N1/H3N2; 1.36 × 10^6^ pfu/ml), constant concentration (2 μg/well) of recombinant HA or NA or decreasing concentration of human properdin (5, 2.5 or 1.25 µg/well) in a 96-well plate using Carbonate-Bicarbonate (CBC) buffer, pH 9.6 at 4°C overnight. After washing out the excess CBC buffer with PBS, three times, 20 µl of concentrated H1N1 or H3N2 (1.36 × 10^6^ pfu/ml) or decreasing concentration of properdin (5, 2.5 and 1.25 µg/ml) was added to corresponding wells, followed by incubation at 37°C for 2h. The binding was detected using respective primary antibodies (1:5000); monoclonal anti-influenza virus H1 HA, A/California/04/2009 (H1N1) pdm09, Clone 5C12 (produced *in vitro*) (BEI-Resources, NIAID, NIH, USA), monoclonal anti-influenza A virus neuraminidase, Clone NA2-1C1 (produced *in vitro*, BEI-Resources, NIAID, NIH,USA, NR-50239) polyclonal anti-influenza virus H3 HA, A/Hong Kong/1/1968 (H3N2) [(antiserum, Goat) (NR-3118 BEI-Resources, NIAID, NIH, USA)], polyclonal anti-VSV-G (1 mg/ml of purified IgG; Imperial College London), or rabbit anti-human properdin (MRC immunochemistry unit, Oxford). After 3 washes with PBS-Tween 20 (0.05%) (PBST), the binding was detected using respective secondary antibodies (1:5000); goat anti-mouse IgG HRP or goat-anti-rabbit IgG HRP (Promega). The colour was developed by adding 3,3’,5,5’-Tetramethylbenzidine (TMB) substrate (50 µl/well). The reaction was terminated using 1M H_2_SO_4_ (50 µl/well), and absorbance was read at 450 nm using a Biorad ELISA plate reader.

For far-western blotting, a 12% v/v SDS PAGE gel with whole virus lysates and recombinant viral envelope proteins were transferred to a PVDF membrane, followed by incubation with properdin (20 μg/ml) for 1h at 4°C, and 1h at room temperature. After three washes (10 mins each) with PBST, the membrane was probed with rabbit anti-human properdin antibody (1:1000), followed by detection with goat-anti-rabbit IgG HRP (1:1000; Promega). The blot was developed using SIGMAFAST™ 3,3′-Diaminobenzidine (DAB) (Sigma) as per the manufacturer’s instructions.

### Infection Assay and qRT-PCR Analysis

A549 cells (0.5 × 10^6^) were seeded overnight in growth media. The next day, IAV particles (MOI 1) were pre-incubated with properdin (20 μg/ml) for 2h at room temperature and added to serum starved A549 cells. Post incubation at 2h and 6h, the cells were washed with PBS gently and pelleted. GenElute™ Mammalian Total RNA Miniprep Kit (Sigma) along with DNase I (Sigma) treatment was used to extract total RNA without any DNA contaminants. The extracted mRNA was converted to cDNA using TaqMan™ High Capacity RNA to cDNA Kit (Applied Biosystems™) and stored at −80°C until needed. qRT-PCR was performed using a StepOnePlus System (Applied Biosystems™) at 95°C for 5 min, followed by 45 cycles of 95°C for 10 s, 60°C for 10 s, and 72°C for 10 s. The relative expression (RQ) was calculated using A549 cells infected with respective viruses as the calibrator. The RQ value was calculated using the formula: RQ = 2^−ΔΔCt^. Primers for each target gene were generated for specificity using the Primer-BLAST software (Basic Local Alignment Search Tool) (36). qRT-PCR was performed using primers listed in **Table 2**; human 18S rRNA was used as an endogenous control to normalise gene expression.

**Table 2:** Forward and reverse primers used for qRT-PCR.

Target	Forward primer	Reverse primer
18S	5′-ATGGCCGTTC TTAGTTGGTG-3′	5′-CGCTGAGCCA GTCAGTGTAG-3′
IL-6	5′-GAAAGCAGCA AAGAGGCACT-3′	5′-TTTCACCAGG CAAGTCTCCT-3′
IL-12	5′-AACTTGCAGC TGAAGCCATT-3′	5′-GACCTGAACG CAGAATGTCA-3′
TNF-α	5′-AGCCCATGTT GTAGCAAACC-3′	5′-TGAGGTACAG GCCCTCTGAT-3′
M1	5′AAACATATGTCTGATAAC GAAGGAGAACAGTTCTT-3′	5′GCTGAATTCTACCT CATGGTCTTCTTGA-3′
RANTES	5′-GCGGGTACCAT GAAGATCTCTG-3′	5′-GGGTCAGAATC AAGAAACCCTC-3′
IFN-α	5′-TTT CTC CTG CC T GAA GGA CAG-3′	5′-GCT CAT GAT TTC TGC TCT GAC A-3′

### Luciferase Assay

Matched H1N1 or unmatched H3N2 pseudotyped particles were pre-incubated with properdin (20 μg/ml) for 2 h at RT. Serum starved MDCK cells (1 × 10^5^ cells/well) were transduced with properdin-preincubated H1N1 or H3N2 pseudotyped particles for 24h in infection medium. IAV pseudotyped particles without properdin preincubation were used as controls. Post incubation, the unbound pseudoparticles were removed, and complete media was added to the cells, followed by incubation for a further 72h for luciferase expression. Luciferase reporter activity was measured using Promega™ Bright-Glo™ Luciferase Assay System (Promega) and Luciferase Plate reader (Promega).

For measuring NF-κB activity, A549 cells (1 × 10^4^ cells/well) were seeded in 12-well plate and transfected with pNF-κB-LUC plasmid using BioT transfection kit (Bioland Scientific) as per the manufacturer’s instructions for 48h. Viral particles (MOI 1), pre-incubated with properdin (20 μg/ml) for 2h, were used to challenge the transfected A549 cells in infection media for 6h. Virus particles that were not treated with properdin were used as control. A549 cells treated with recombinant TNF-α (10 ng/ml) and IL-1β (10 ng/ml) (R&D Systems) was used as a positive control for NF-κB activation. Luciferase reporter activity was measured as described above.

### Cell Binding Assay

Cell binding assay was performed to evaluate if the binding of the IAV virions with properdin affected the ability of the IAV virions to bind to the cell surface. Briefly, A549 cells (1 × 10^5^ cells/well) were challenged with H1N1 or H3N2 (1.36 × 10^6^ pfu/ml) viruses, pre-incubated with or without properdin (20 µg/ml) and incubated at 37°C for 2h. Following PBS washes, the cells were fixed with 1% v/v paraformaldehyde (Fisher Scientific) for 1 min at room temperature, followed by blocking with 2% w/v BSA for 2 h at 37°C. The binding was probed with either polyclonal anti-influenza virus H3 (1:5,000; BEI-Resources), or monoclonal anti-influenza H1 (1:5,000; BEI-Resources). The colour was developed by adding TMB substrate (50 µl/well) and the reaction was stopped using 1M H_2_SO_4_ (50 µl/well). The absorbance of the plate was read at 450 nm using an ELISA plate reader (BioRad).

### *In-Silico* Docking Analysis

Crystal structures of HA of H1N1 (PDB ID: 4M4Y) and H3N2 (PDB ID: 4FNK) in trimeric form, NA of H1N1 (PDB ID: 6Q23) and H3N2 (PDB ID: 4GZX) in tetrameric form, and human properdin (PDB ID: 6S0A) were retrieved from RCSB PDB database. The 3D structures of HA lacked the transmembrane region, and that of NA lacked the stalk and transmembrane region. The cleavage site of HA is known to be essential for adopting the active conformation (37). These residues were found to be missing in 4M4Y and 4FNK crystal structures. The missing cleavage site residues (ILE326, GLN327, SER328, ARG329 of 4M4Y and GLN327, THR328, ARG329 of 4FNK) were modelled *de novo* using a random tweak algorithm (38).

The above structures were cleaned to remove water molecules and other artefacts, protonated, minimised and typed with CHARMm forcefield (39).

HA and NA structures of H1N1 and H3N2 virus were docked with human properdin using the ZDOCK algorithm (40) available in BIOVIA Discovery studio release 2021 (41). 1000 top poses were selected and clustered using RMSD and interface cutoff of 10 Å. The clusters were sorted according to the cluster size and binding energy (Z-Rank score). The lowest energy poses among the top 10 clusters were selected and analysed for the intermolecular interactions.

### Statistical Analysis

The graphs were generated and statistical significance calculated using the GraphPad Prism 9.0 software. The statistical significance was considered as indicated in the figure legends between treated and untreated conditions. Error bars show SD or SEM as stated in the figure legends.

## Results

### Human Neutrophils Challenged With IAV Release Properdin Which Interacts With IAV-Subtypes

**Figure 1:**
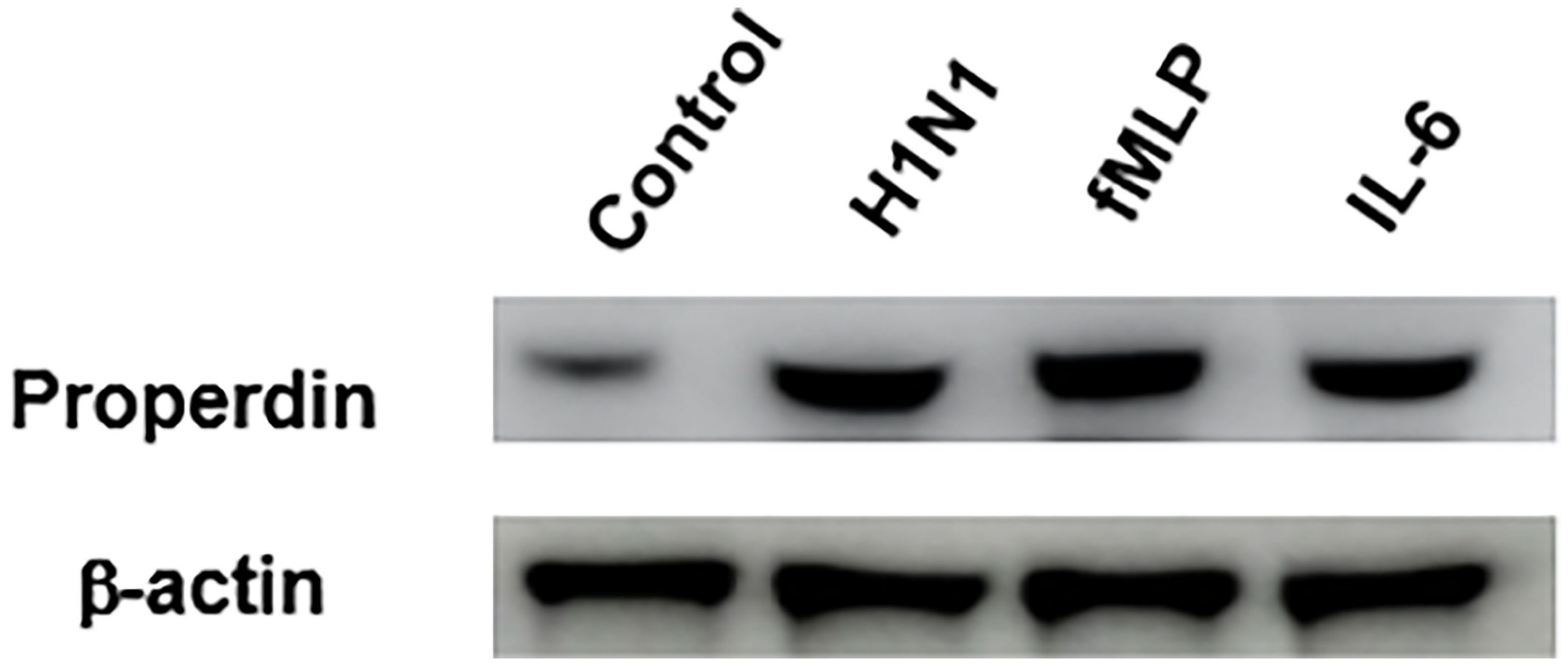
IAV infection induces properdin release from neutrophils. Freshly isolated human peripheral blood neutrophils were challenged overnight with H1N1 (Lane 2). Unchallenged neutrophils were used as control (Lane 1); fMLP (1µM) (lane 3) and IL-6 (10 ng/ml; Sigma) (lane 4) were used as a positive control. The supernatants of the treated neutrophils were collected, concentrated, and subjected to SDS-PAGE separation in 1 x MOPS buffer (4-12%, NuPAGE gel) (40µg total protein/well). 10µg β-actin was added to each treated sample as a loading control prior to SDS-PAGE. Secreted properdin was detected by Western Blotting using rabbit anti-properdin (1: 2000 dilution), and actin was detected by rabbit anti-actin antibody (1:13000 dilution). ECL was performed using standard HRP-labelled secondary antibodies that were exposed for 2 seconds. Source blot for properdin (**Figure S1A**) and β-actin (**Figure S1B**) are provided in the **Supplementary Material**.

The release of properdin by the H1N1-challenged neutrophils were assessed by western blotting (**Figure 1**). A clear difference in properdin levels was observed in IAV H1N1-challenged neutrophils compared to supernatant from the control sample (neutrophils alone). Neutrophils treated with IL-6 or fMLP, two well known inducers of neutrophils, were used as positive controls (**Figure 1**). The amount of properdin produced by H1N1 challenged neutrophils compared well with its positive control, suggesting that the virus was a potent inducer of properdin released from neutrophil granules.

**Figure 2:**
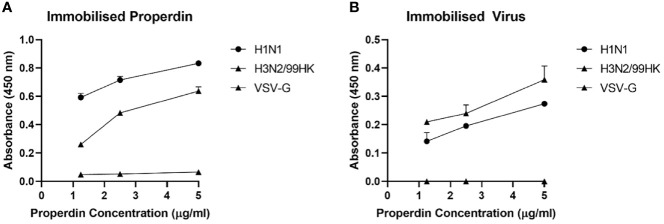
Human properdin bound both IAV-subtypes in a dose-dependent manner. Decreasing concentration of immobilized properdin (5, 2.5 and 1.25 µg/ml) **(A)** or constant concentration of whole virus particles (1.36 × 106 pfu/ml) **(B)** were coated in a 96-well plate using Carbonate-Bicarbonate (CBC) buffer, pH 9.6 at 4°C overnight. After washing out the excess CBC buffer with PBS, three times, 20 µl of concentrated H1N1 or H3N2 (1.36 × 106 pfu/ml) **(A)** or decreasing concentration of properdin (5, 2.5 and 1.25 µg/ml) **(B)** was added to corresponding wells, followed by incubation at 37°C for 2h. The wells were probed with corresponding primary antibodies (1:5000; 100 µ/well); monoclonal anti-influenza virus H1, polyclonal anti-influenza virus H3, or rabbit anti-human properdin, after washing out the unbound H1N1, H3N2 or properdin. VSV-G pseudotyped lentivirus was used as a negative control and was detected using polyclonal anti-VSV-G. The data were presented as a mean of three independent experiments done in triplicates µ SEM.

The ability of immobilised native properdin to bind H1N1 and H3N2 viral particles (**Figure 2A**) and vice versa (**Figure 2B**) was assessed using a direct ELISA (**Figure 2**). Solid-phase properdin bound both IAV subtypes in a dose-dependent manner when probed with either polyclonal anti-influenza virus H3, or monoclonal anti-influenza H1. The immobilised viral particles also bound properdin in a dose-dependent manner when probed with rabbit anti-human properdin but with lower efficiency. Vesicular stomatitis virus G (VSV-G) protein pseudotyped lentivirus particles were used as a negative control RNA virus, which showed no significant binding with properdin at various concentrations tested.

**Figure 3:**
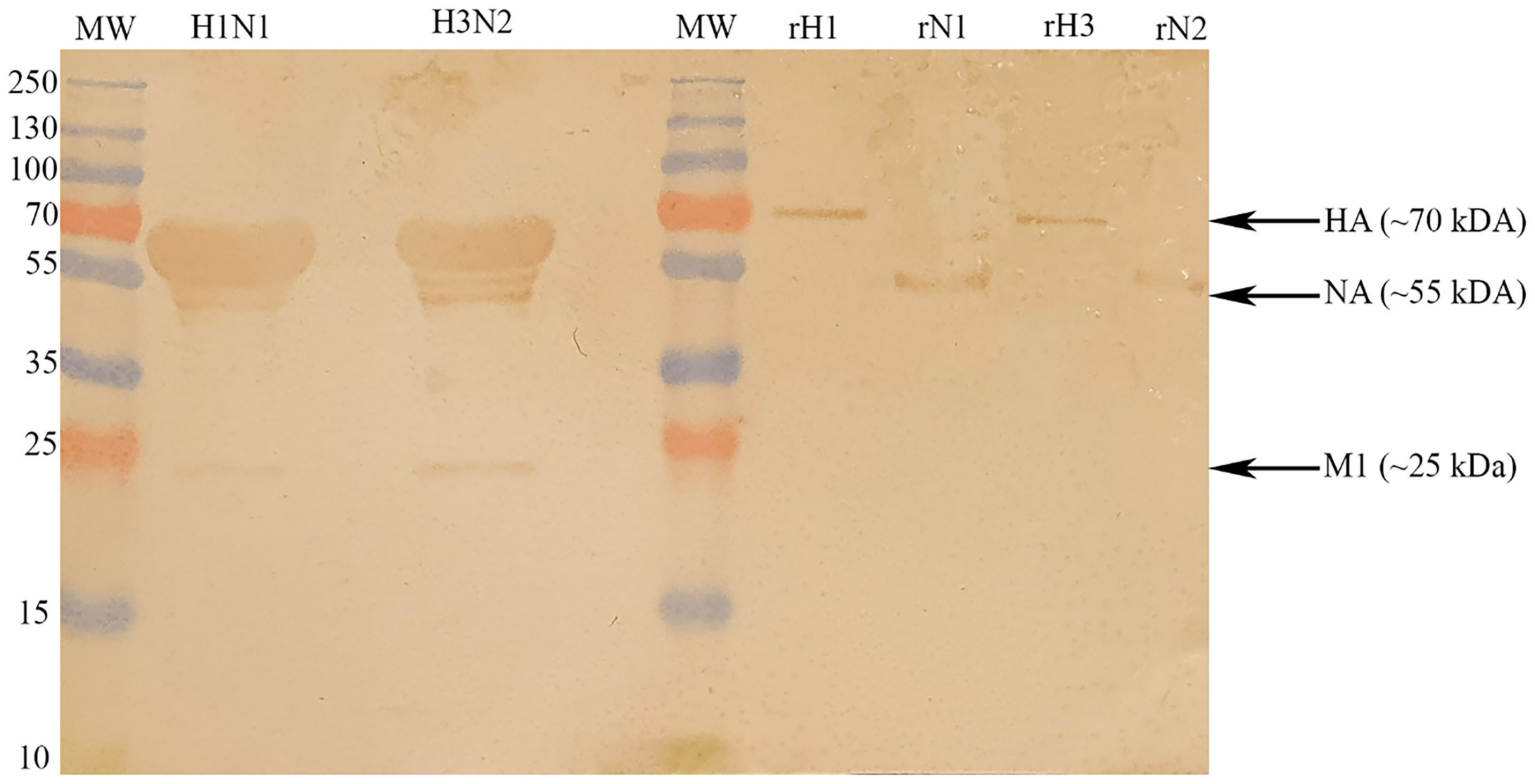
Far-western blotting analysis to show properdin (20 µg/ml) binds HA, NA and M1 of purified H1N1 and H3N2 subtypes. 10 μl of concentrated H1N1 (Lane 2) or H3N2 (Lane 4) virus (1.36 × 10^6^ pfu/ml) was first run on the SDS-PAGE (12% w/v) under reducing conditions and then transferred onto a nitrocellulose membrane. Following the blocking process with PBS+5% w/v BSA, the membrane was incubated with 20 μg/ml of properdin. After PBS washes, the membrane was probed with rabbit anti-human properdin antibody (1:1000). Properdin bound to M1 (∼25 kDa), HA (∼70 kDa) and NA (∼55 kDa) in the case of both pH1N1 and H3N2 subtypes. In the same blot, the identities of properdin bound IAV glycoproteins were validated using recombinant HA and NA.

**Figure 4:**
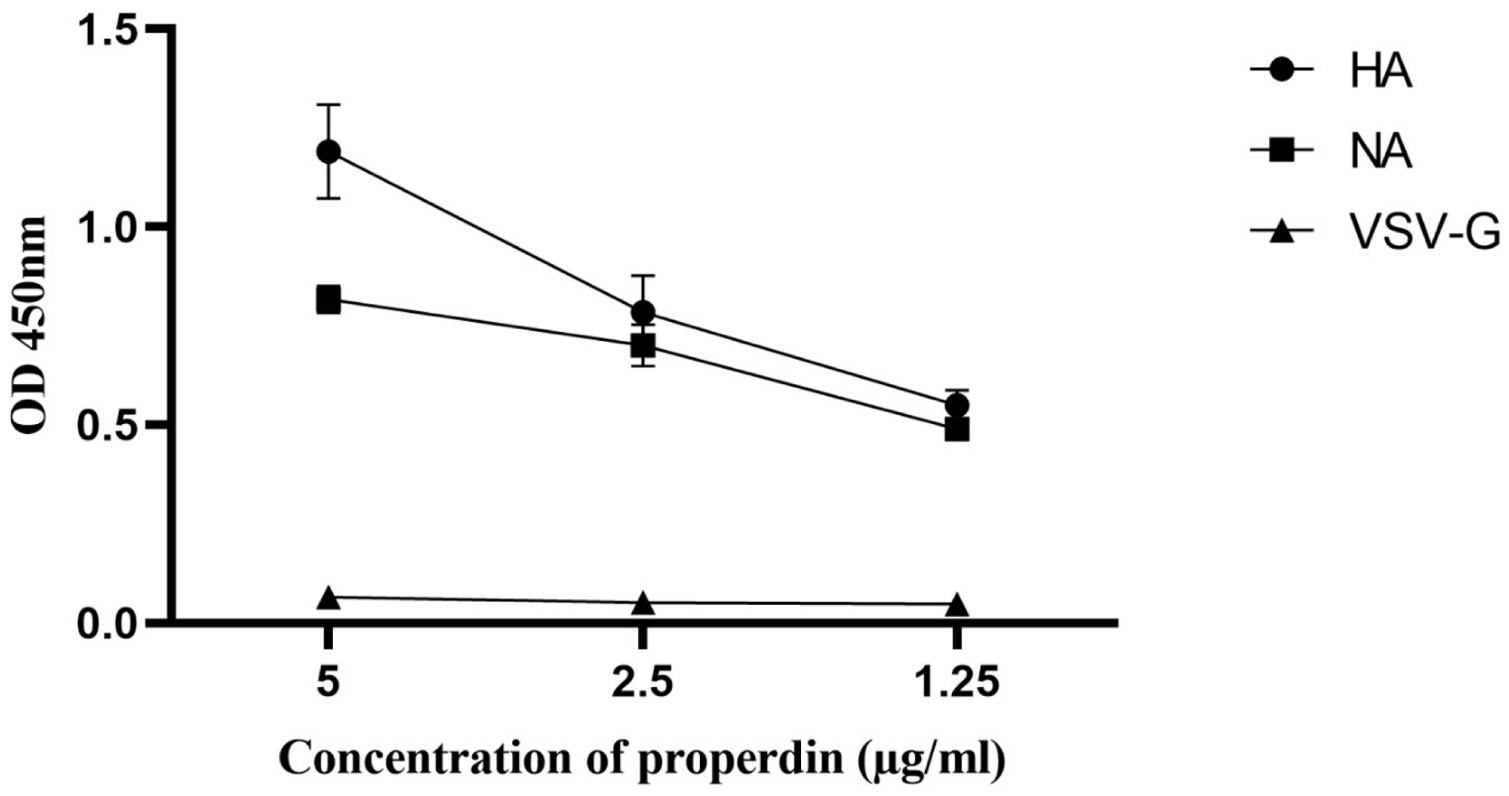
Direct ELISA to show the ability of properdin to bind purified recombinant hemagglutinin (HA) and neuraminidase (NA) of H1N1. Microtiter wells were coated with varying concentrations of properdin (5, 2.5, and 1.25 μg/ml). 2 µg/well of recombinant HA or NA was diluted in 200 μl of PBS and was added to all the wells. After washes with PBS, the binding was probed with either monoclonal anti-influenza virus H1, monoclonal anti-influenza A virus neuraminidase or polyclonal anti-VSV-G antibody. VSVG was used as a negative control protein, where no significant binding was detected. The data were expressed as the mean of three independent experiments carried out in triplicates ± SEM.

Far-western blotting analysis was performed to determine properdin (20 µg/ml) binding to IAV glycoproteins (**Figure 3**). Properdin was found to bind surface glycoproteins, hemagglutinin (HA; ~70 kDa) and neuraminidase (NA; ~55 kDa), in addition to matrix protein 1 (M1; ~25 kDa) (**Figure 3**). Additionally, an ELISA was performed to quantitatively validate properdin binding to a constant concentration of purified recombinant H1N1 HA (2 µg/well) and H1N1 NA (2 µg/well) (**Figure 4**). Properdin bound HA and NA in a concentration-dependent manner; properdin bound purified HA better than NA (**Figure 4**). Again, no significant binding was observed for VSV-G pseudotyped lentivirus particles.

### Human Properdin Modulates Infection of A549 Lung Epithelial Cells

**Figure 5:**
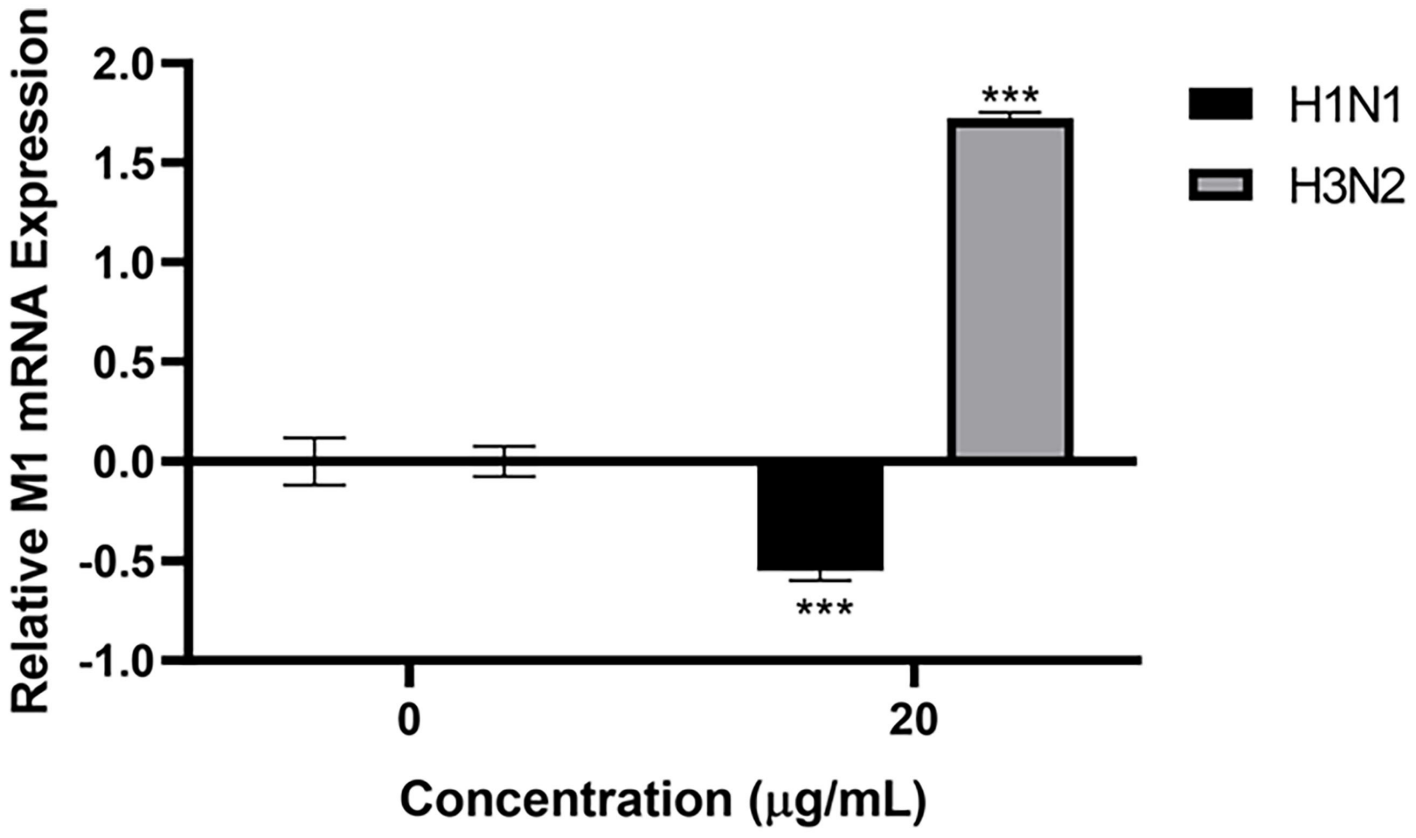
Properdin treatment suppresses infection and replication of H1N1-infected A549 cells, while up-regulation of M1 replication was seen with H3N2. mRNA transcript levels of M1 expression of both pH1N1 and H3N2 IAV subtypes (IAV) (MOI 1) after infection of A549 cells at 6 h were measured. A549 cells were incubated with H1N1 or H3N2, pre-treated with or without human properdin (20 μg/ml). Following cell lysis, RNA was extracted and converted into cDNA. Infection was measured *via* qRT-PCR using M1 primers; 18S was used as an endogenous control. Data shown are normalised to M1 levels of respective untreated control (cells + virus only). Significance was determined using the two-way ANOVA test (***p < 0.001) (n = 3).

HA, NA and M1 are crucial proteins of IAV, which have many essential functions, including viral entry, budding of virions, IAV infectivity and replication. Since human properdin bound to HA, NA, and M1 of IAV, an infection assay was performed to assess the ability of IAV subtypes to infect A549 lung epithelial cells in the presence of properdin (20 µg/ml) (**Figure 5**). A549 cells challenged with properdin pre-treated IAV subtypes showed differential expression of M1 mRNA transcript levels. In the case of H1N1, properdin pre-treatment down-regulated M1 expression by -0.5 log_10_ at 6h compared to untreated cells (Cells + H1N1), suggesting that the properdin interaction with H1N1-viral particles reduced the viral replication efficacy at an early stage of the IAV-replicative cycle. However, H3N2-infected A549 cells, pre-treated with properdin, showed an up-regulation (1.7 log_10_) of M1 expression, suggesting an enhanced replication (**Figure 5**).

### Modulation of Immune-Response in IAV-Infected A549 Cells by Properdin

**Figure 6:**
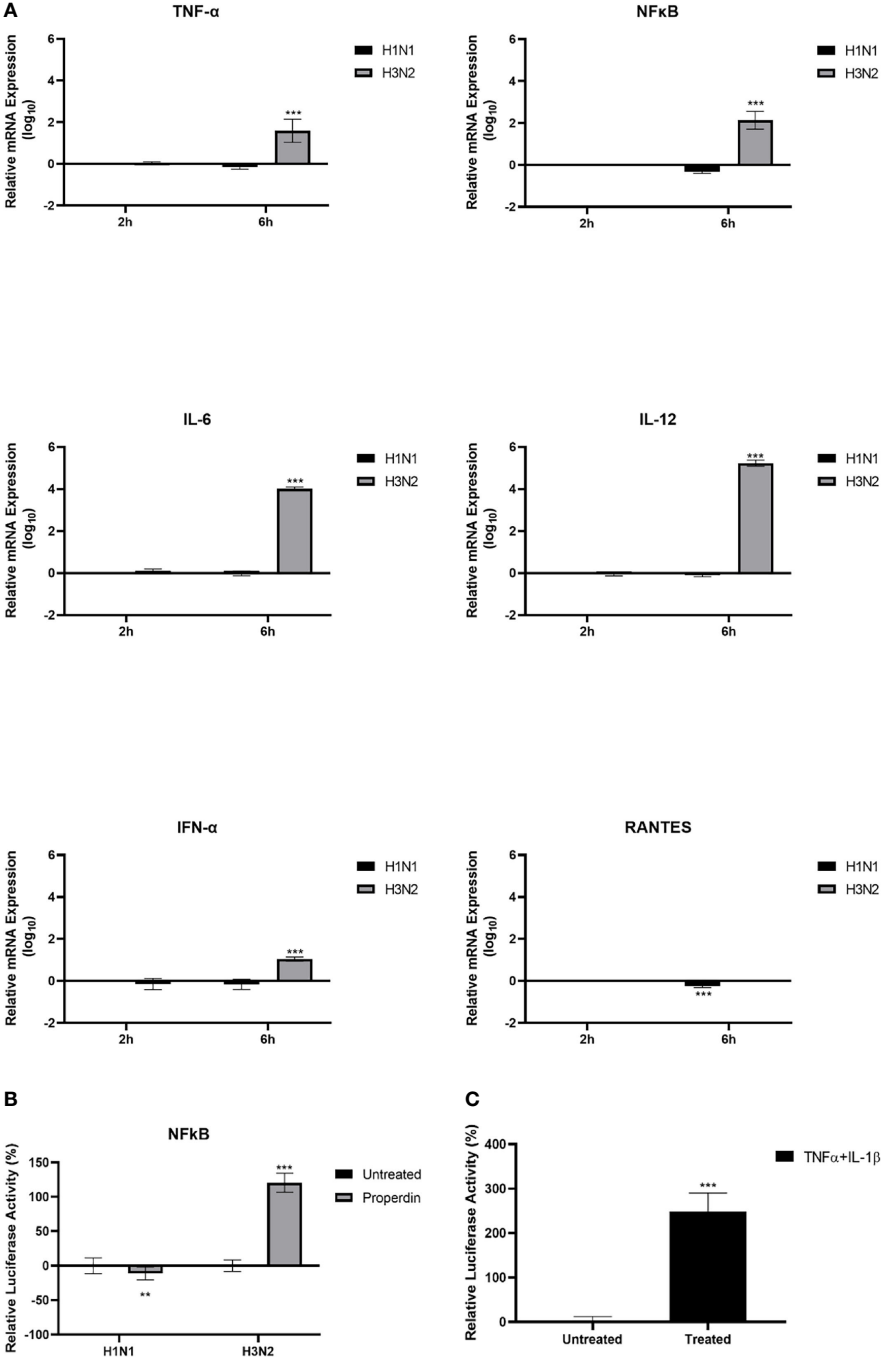
Properdin triggers an anti-inflammatory response in H1N1-infected A549 cells while provoking a pro-inflammatory response in the case of H3N2 infection. mRNA expression levels of selected cytokines and chemokines (TNF-α, IL-12, IL-6, RANTES, IFN-α and NF-κB) were measured using qRT-PCR using the primers listed in **Table 2**. **(A)** The data were normalised *via* 18S rRNA expression as an endogenous control. The relative expression (RQ) was calculated using the untreated sample (cells + virus only) as the calibrator. The RQ value was calculated using the formula: RQ = 2−ΔΔCt. NF-κB activation was measured *via* luciferase reporter assay in these cells following challenge with properdin treated H1N1 or H3N2 subtypes **(B)**. The relative NF-κB activity was calculated using the untreated sample (cells + virus only) as the baseline. A549 cells treated with TNF-α and IL-β was used as a positive control for NF-κB activation **(C)**. Assays were conducted in triplicates, and error bars represent ± SEM. Significance was determined using the two-way ANOVA test (**p < 0.01 and ***p < 0.001) (n = 3).

The mRNA levels of pro-inflammatory cytokines and chemokines in IAV-infected A549 cells with and without properdin (20 µg/ml) treatment were determined by qRT-PCR at 2h and 6h time points (**Figure 6A**). The relative mRNA expression levels of IL-12, IL-6, TNF-α, and RANTES were down-regulated following properdin treatment at 6h in H1N1-infected A549 cells. However, in the case of H3N2-infected cells, properdin treatment caused up-regulation of mRNA levels of IL-12, IL-6, TNF-α and RANTES, suggesting that properdin affected these cells in an IAV-subtype dependent manner (**Figure 6A**). During IAV infection, IFN-α is also one of the critical type I interferon that possesses strong antiviral and immune-modulatory properties (42). H1N1-infected A549 cells pre-treated with properdin showed a reduction in mRNA levels of IFN-α, while upregulation was observed in the H3N2-infected cells. This suggests the ability of properdin to reduce the efficacy and rate of viral replication, thus, diminishing the levels of IFN. Furthermore, nuclear factor kappa B (NF-κB), a nuclear transcription factor, is activated by IAV infection, thereby causing the overexpression of IAV proteins, including HA, NA and M1, during viral infections (43–45). In this context, the NF-κB mRNA expression was also measured in IAV-infected A549 cells following properdin treatment (**Figure 6A**). Properdin treatment downregulated NF-κB levels (-0.5 log_10_) in H1N1-infected cells at 6h while promoting the expression in H3N2-infected cells by 2.3 log_10_. This NF-κB suppression can be attributed to either a reduced viral titre in the case of H1N1-infected A549 cells or the possibility of IAV-NS1 acting as a suppressor of NF-κB activation (46). Furthermore, luciferase reporter assay was also performed to determine the NF-κB activation in IAV-infected A549 cells treated with and without properdin levels (**Figure 6B**). The data was consistent with the qRT-PCR analysis; a reduced NF-κB luciferase reporter activity (-11%) was observed in A549 cells infected with H1N1 pre-incubated with properdin, while enhanced activity (approximately ~3-fold) was detected in the case of H3N2 (**Figure 6B**). A549 cells treated with recombinant TNF-α and IL-1β were used as a positive control (**Figure 6C**), which showed a significant increase in NF-κB levels.

### Properdin Treatment Reduces Luciferase Reporter Activity *Via* Pseudotyped Viral Particles

**Figure 7:**
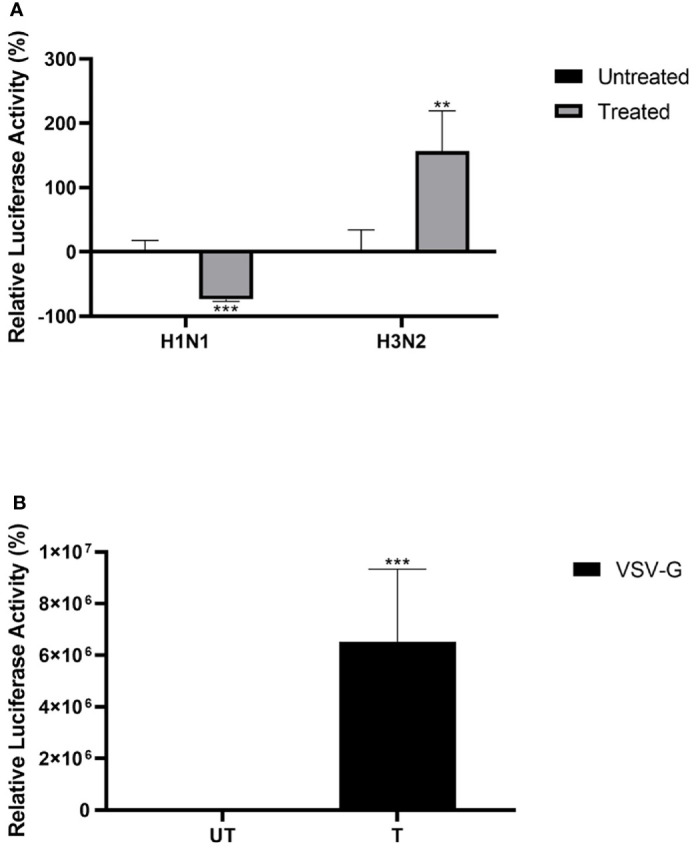
Properdin treatment modulates IAV entry in a subtype-dependent manner. Matched H1N1 or unmatched H3N2 pseudo-typed lentiviral particles were pre-treated with properdin (20 μg/ml) **(A)**. Luciferase reporter activity of MDCK cells transduced with either treated or untreated pseudo-typed lentiviral particles was measured to study if the treatment affected the ability of the virus to enter the cells. VSV-G pseudotyped lentiviral particles were used as positive control **(B)**. The background was subtracted from all data points. The data obtained were normalised with 0% luciferase activity being defined as the mean of the relative luminescence units recorded from the control sample (Cells + respective pseudotyped lentiviral particle). Data are shown as the normalized mean of three independent experiments done in triplicates ± SEM. Significance was determined using the two-way ANOVA test (**p < 0.01, ***p < 0.001) (n = 3).

Lentiviral pseudotypes were generated to study the effect of properdin on the viral cell entry independent of other IAV components of H1N1 and H3N2 subtypes (**Figure 7**). Approximately 73% reduction in luciferase reporter activity was observed in MDCK cells infected with H1N1-lentiviral pseudotypes pre-incubated with properdin (20 µg/ml), compared to untreated control (Cells + H1N1 lentiviral pseudotypes) (**Figure 7**). However, an opposite effect was observed in the case of MDCK cells challenged with H3N2-lentiviral pseudotypes pre-incubated with properdin; ~2.5 -fold increase in luciferase reporter activity was seen. Thus, properdin acts as an entry inhibitor against the H1N1 subtype while it promotes entry of the H3N2.

### The Initial Stage of IAV Infection Is Inhibited by Properdin

**Figure 8:**
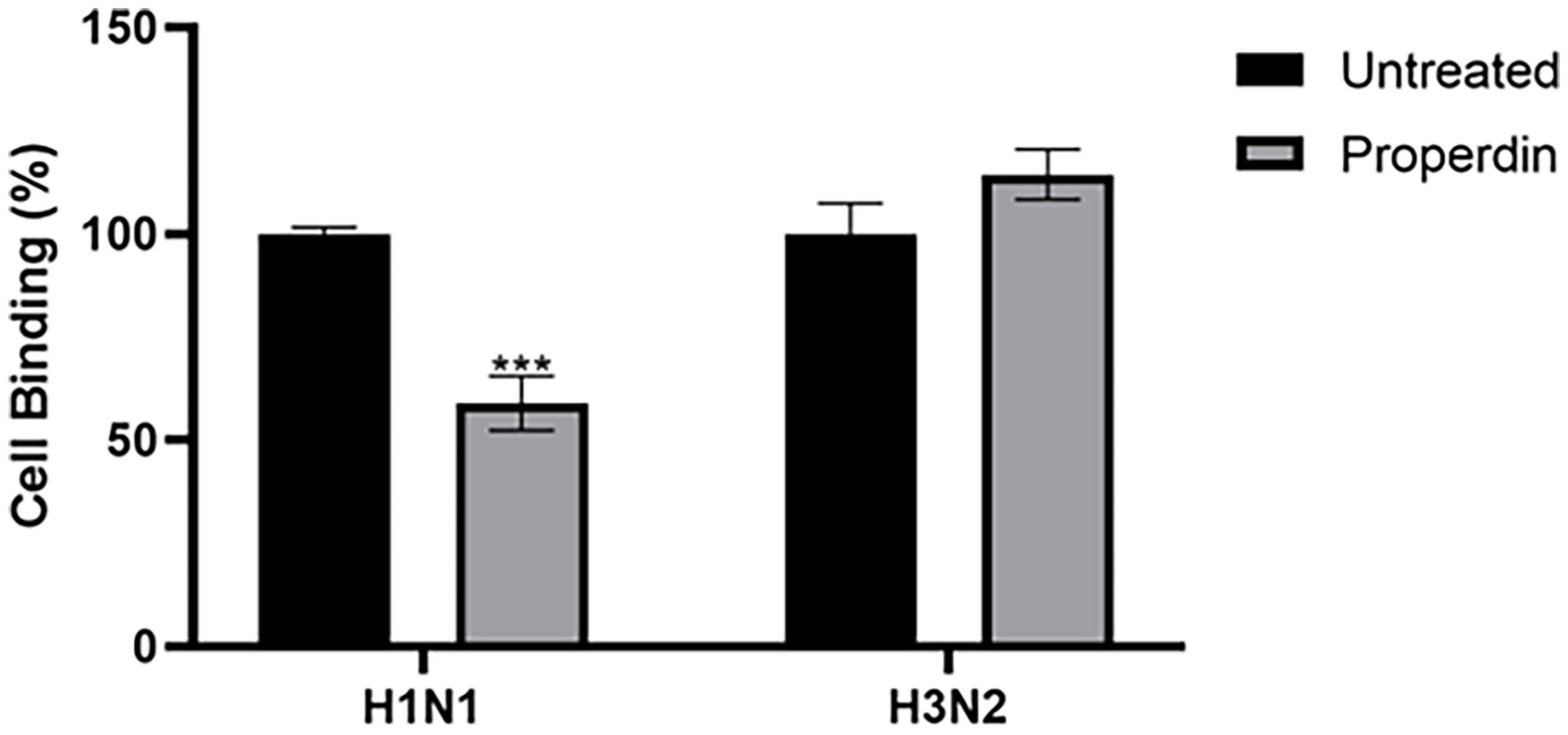
Cell binding assay to show properdin treatment inhibits IAV infection by restricting H1N1 or H3N2 viral particles binding to the cell-surface receptor. A549 (1 × 10^5^ cells/ml) cells were infected with H1N1 or H3N2 viral particles pre-incubated with or without properdin (20 µg/ml), followed by incubating at 37°C for 2h. After removing unbound protein and viral particles, the wells were fixed with 1% v/v paraformaldehyde for 1 min and probed with corresponding primary antibodies; monoclonal anti-influenza virus H1 or polyclonal anti-influenza virus H3 antibodies (1:5000). Three independent experiments were carried out in triplicates, and error bars express as ± SEM. Significance was determined using the two-way ANOVA test (***p < 0.001) (n = 3).

Reduced luciferase activity in the H1N1 subtype treated with properdin (20 µg/ml) suggested that properdin might block IAV infection at the early stage of the IAV cycle. A cell-binding assay was performed to assess if properdin pre-treatment inhibited viral infection by preventing the binding of IAV virions to the A549 cell surface (**Figure 8**). In this assay, ~40% reduction in cell binding was observed in properdin pre-treated and H1N1 challenged A549 cells. In comparison, ~20% increase in cell binding was noted with A549 cells challenged with H3N2 pre-incubated with properdin compared to cells infected with virus alone. Reduction in cell binding following properdin treatment in the case of H1N1-challenged A549 cells highlighted that properdin prevented the viral particles from binding to their cell surface receptors and thus, acting as an entry inhibitor against the H1N1 subtype.

### Properdin Interacts With HA and NA of H1N1 With a Higher Binding Affinity as Compared to H3N2

**Figure 9:**
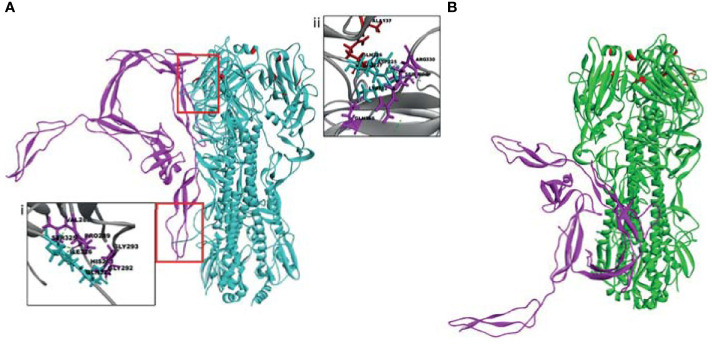
The top docked poses indicate that human properdin (pink) binds to the sialic acid-binding site (upper red box) and cleavage site (lower red box) of HA of **(A)** H1N1 and not of **(B)** H3N2. Docking of human properdin to HA receptors of H1N1 and H3N2 was performed using the Z-Dock algorithm. The lowest energy pose from the top 10 clusters was selected as the top pose. Intermolecular interactions between receptor (HA) and ligand (Properdin) (< 10 Å distance) were analysed. Hydrogen bonds (dotted line) between HA of H1N1 and properdin at the cleavage site is shown in the inset (i) between receptor-ligand residues SER325 - VAL288, ILE326 - PRO289, GLN327 - GLY292, GLN327 - GLY293; and with residues SER325, ARG330 proximal to the sialic acid receptor binding site in the inset (ii). Sialic acid receptor binding site residues are depicted in red.

*In-silico* docking analysis was performed to compare the binding affinity of properdin to HA and NA of H1N1 and H3N2. The analysis indicated that properdin could interact with HA and NA of both the subtypes. Properdin was found to exhibit a higher binding affinity towards HA and NA of H1N1 than H3N2, as inferred from the binding energy scores (**Table 3**). Analysis of the top docked poses revealed that properdin engaged in the hydrogen bond interactions with three residues of the cleavage site - SER325, ILE326, GLN327 present in HA of H1N1, while a weak hydrophobic interaction involved with LYS326 present at the cleavage site of HA of H3N2 in the top docked pose (**Table 3**; **Figure 9A**). Properdin was also found to interact with the residues (LYS222, ASP225, GLU227) proximal to the sialic acid receptor binding site (GLN226, THR136, ALA137, HIS183, ASP190) (47) in HA of H1N1. These interactions were absent in the top docked pose of properdin with HA of H3N2 (**Figure 9B**).

**Table 3:** Binding energy of properdin with IAV receptors (HA and NA of H1N1 and H3N2) and their interacting residues from the lowest energy docked poses.

IAV type	Receptor (PDB ID) – ligand (PDB ID) complex	Binding energy (kcal/mol)	Receptor - ligand interacting residues
H1N1	**Hemagglutinin (4M4Y) – properdin (6S0A)**	-99	*Hydrogen bond interaction*: LYS166 - MET420, LYS166 - VAL421, LYS242 - GLN343, THR25 - PRO265, LYS46 - ASN307, **ILE326** - PRO289, **GLN327** - GLY293, ASP225 - SER325, GLU238 - GLN364, LYS222 - GLN365, ASN21 - ASN285, THR23 - GLN281, SER91 - CYS350, SER91 - CYS350, LYS222 - ILE328, ASN21 - GLY261, **SER325** - VAL288, **GLN327** - GLY292, GLU227 - ARG330, ASP271 - GLY352
			*Electrostatic interaction*: GLU227 - ARG330, ASP271 - LYS354
			*Hydrophobic interaction:* PRO128 - VAL421, PRO90 - LYS354, LYS313 - PRO268, PRO324 - VAL288, **ILE326** - PRO289, LYS46 - ALA309, ILE219 - ARG330, PRO90A - ARG353, HIS47 - VAL310, HIS47 - PRO311, **ILE326** - PHE295
H3N2	**Hemagglutinin (4M4Y) – properdin (6S0A)**	-78	*Hydrogen bond interaction:* ASN54 - THR308, LYS315 - GLU323, LYS315 - GLU323, GLU61 - PHE355, SER45 - GLN364, SER46 - GLN365, ALA19 - ARG434, ALA19 - ARG434, ASN53 - GLN110, THR30 - LYS114, PRO55 - ALA309, GLU61 - PHE355, GLN33 - GLY113
			*Electrostatic interaction:* LYS264 - ASP356, ASP32 - ARG351, ASP19 - LYS446, ASP31 - LYS114
			*Hydrophobic interaction:* PRO55 - VAL310, **LYS326** - PRO393, ILE18 - LEU436, ILE278 - ALA309
H1N1	**Neuraminidase (6Q23) - properdin (6S0A)**	-107	*Hydrogen bond interaction:* SER82 - GLU146, ASN450 - FUC1, SER82 - SER181, NAG502 - ASN164, SER391 - CYS89, ASP416 - GLY137, LEU127 - GLN179, ASN450 - FUC1, SER82 - GLU146, CYS92 - GLY172, PRO93 - CYS170, SER95 - FUC1, PRO380 - ASP129, GLU412 - ARG161, LEU415 - CYS174, NAG502 - ASN164, NAG502 - ASN164, THR413 - GLN177
			*Electrostatic interaction:* SER82 - ASP185, SER82 - THR186, SER82 - GLU146
			*Hydrophobic interaction:* CYS417 - HIS173, CYS417 - PRO168, LEU127 - ALA183, PRO93 - FUC1, ARG172 - FUC205, LEU415 - HIS173
H3N2	**Neuraminidase (4GZX) - properdin (6S0A)**	-92	*Hydrogen bond interaction:* ARG283 - VAL271, ARG288 - VAL271, ARG288 - THR272, ASN306 - FUC1, ILE312 - PHE355, GLY357 - SER267, ASN358 - ILE305, ASP355 - CYS269, SER384 - ASN307, SER266 - ASP356, SER311 - ASP356, SER311 - ASP356, GLY357 - SER267, TRP383 - ASN307, SER384 - ASN307, ASP359 - PRO265, GLN91 - PRO268, ILE312 - PHE355, ASP309 - HIS358
			*Hydrophobic interaction:* VAL313 - VAL310, VAL313 - PRO311, VAL313 - PRO313, VAL313 - CYS273, CYS318 - ALA309, CYS337 - ALA309, ILE302 - VAL310, VAL313 - FUC1, VAL313 - PHE355

The residues in bold depict the cleavage site residues of HA. Underlined residues are proximal (within 10 Å) to the sialic acid receptor binding domain of HA.

**Figure 10:**
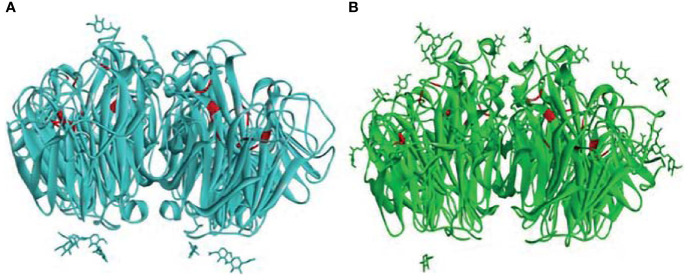
Comparison of the glycosylation patterns as observed in the crystal structures of **(A)** H1N1 NA (PDB ID: 6Q23) and **(B)** H3N2 NA (PDB ID: 4GZX) receptors. The sialic acid binding site residues ARG118, ASP151, ARG152, ARG224, GLU276, ARG292, ARG371, TYR406 (48) in the two NAs are depicted in red.

The top docked poses of properdin with HA of H1N1 and H3N2 revealed proximal binding sites. However, this was not the case for properdin docked with NA of H1N1 and H3N2. This may be due to the lack of availability of complete crystal structures of NA of H1N1 and H3N2. In addition, differences in glycosylation patterns were evident in the NA crystal structures of H1N1 and H3N2. In H3N2 NA, the glycosylation sites were present near the sialic acid-binding sites, unlike in H1N1 NA, where the glycosylation sites were present near the stalk region (**Figure 10**). The different overexpression systems used (*Spodoptera frugiperda* for NA of H1N1 and *Trichoplusia ni* for NA of H3N2) for elucidating the crystal structures of H1N1 and H3N2 NA could have led to the glycosylation differences observed in these structures. In the absence of the complete crystal structures of NA of H1N1 and H3N2 and the distinctly dissimilar glycosylation patterns observed in these two structures, it is difficult to be certain about inference based on the docking experiments of properdin and NA of H1N1 and H3N2.

## Discussion

As a critical component of innate immunity, the complement system is an essential cascade for protection against numerous microbial infections, including viruses. The complement components employ several mechanisms that can restrict the viral infection of the host (2, 49). However, many infectious viral pathogens have been found to actively target the complement components as a defensive strategy to replicate and spread more efficiently (2, 49, 50).

Properdin, the only known up-regulator of the complement alternative pathway, interacts with surface-bound C3b and recruits more C3b to form and stabilise the alternative pathway C3 convertase complex, C3bBb. In addition, properdin has also been proposed to act as a pattern recognition molecule for initiating alternative pathway. Properdin is reported to bind to the non-self surfaces such as pathogens, cancer cells, and nanoparticles stimulating C3 convertase formation (51) as well as opsonisation (52, 53). Properdin is also known to directly interact with Glycosaminoglycans (54), including heparin (55), heparan sulfate (56, 57), dextran sulfate, fucoidan (58), chondroitin sulfate (57), and DNA on late apoptotic and necrotic cells (21). Studies have highlighted properdin’s ability to act as a pattern-recognition receptor (PRR) for *Neisseria gonorrhoeae* (59), *Salmonella typhimurium* lipopolysaccharide (LPS), *Neisseria meningitidis* lipooligosaccharide (7), and *Chlamydia pneumoniae* (60). Properdin can also bind gp41 and gp120, subunits of the HIV-1 envelope (24), and may prevent HIV-1 from binding to the CD4 receptor. Thus, the properdin-HIV-1 interaction may restrict the fusion of the viral envelope with the cell membrane (24). Properdin is also reported to act as an opsonin. Recombinant properdin was found to enhance the opsonization of *N. meningitidis* and *S. pneumonia in vitro*  *(* 61). Additionally, properdin was also reported to act as an opsonin for carbon nanotubes without involving complement recruitment and activation (53). The opsonisation was found to enhance the uptake and clearance of the nanoparticles, in addition to upregulating pro-inflammatory cytokines/chemokines in THP-1 cells, (a macrophage cell line) (53).

*In vivo* studies using animal models have demonstrated the role of the complement system in protecting against seasonal IAV infection (62–64). IAV infection is a major respiratory tract infection in humans responsible for a higher morbidity outcome (five million cases per year) with up to half a million mortality rate worldwide (65). During IAV infection, immune cells, including neutrophils, are known to increase in number. Neutrophils can recognise viruses *via* PRRs as either opsonised virions or endosomal toll-like receptors (TLRs). Neutrophils can respond to viral pathogen-associated molecular patterns (PAMPs) with respiratory burst, degranulation of proteases and cytokines, and/or NETosis. In mice, humans and ferrets, pathogenic IAV infection causes an increase in neutrophils in the lungs and blood (66–68). During severe influenza pneumonia and highly pathogenic avian influenza infections, the degree of neutrophil infiltration in the lower respiratory tract has been linked to illness severity (69). Neutrophils are also among the initial responders to IAV infection in the lungs. Neutrophils are known to have sialic acid receptors and are themselves susceptible to IAV infection. IAV infection increases apoptosis in neutrophils, although infection does not result in viral replication. TLRs that detect nucleic acids are abundant in human neutrophils, particularly endosomal TLR-8 (70, 71). RIG-like receptors (RLRs) are also found in neutrophils and aid in detecting viral dsRNA (72). The activation of these TLRs and RLRs by IAV can cause neutrophil degranulation. Neutrophils produce intracellular granules in a specified order (azurophilic, specific, gelatinase, secretory) and secrete granules in the exact opposite manner (73). Properdin is released from the specific granules of activated peripheral blood neutrophils, which can directly interact with pathogens (50, 74). As shown in **Figure 1**, H1N1 challenge to the neutrophils increased the amount of properdin released when compared to the unchallenged neutrophils; the amount released matched with that produced by IL-6 challenge, suggesting that IAV on its own can induce properdin release by neutrophils without requiring any pro-inflammatory cytokine.

Furthermore, an important message arising out of this study is the ability of properdin to bind IAV glycoproteins, including HA and NA, in addition to M1. As the M1 protein lies beneath the lipid layer, the interaction likely occurs by the abundantly present non-packaged M1 that are released from ravaged cells during late stages of infection to help increase the odds of survival of the virus (75). The interaction of properdin with the IAV glycoproteins helps in the suppression of replication in H1N1 infected A549 cells while promoting the replication and infectivity in the case of H3N2 subtype. The effect of properdin on A549 cell viability was assessed to confirm that the differential effects of M1 resulted solely from the interaction of properdin with the viral proteins. As evident in **Supplementary Figure S2**, A549 cells treated with properdin alone were not found to significantly affect cell viability even at longer incubation time, i.e. 24h. Additionally, A549 cells infected with properdin-pre-treated H1N1 or H3N2 subtype showed differential expression of mRNA levels of TNF-α, IFN-α, IL-6, and RANTES. In the case of the H1N1 subtype, properdin treatment resulted in an anti-inflammatory response as evident by suppressed mRNA levels of these cytokines/chemokines at 6h post-infection. However, mRNA levels of these cytokines/chemokines were enhanced in the case of the H3N2 subtype infection, suggesting the trigger of a pro-inflammatory response. In this study, treatment of A549 cells with properdin alone was not found to significantly affect the mRNA expression levels of cytokines studied at the time points tested (**Supplementary Figure S3**). In a previous study by Al-Mozaini et al., human properdin was found to cause an increase in pro-inflammatory cytokine expression (TNF-α, IL-1β, and IL-6) in THP-1 cells infected with *M. bovis* BCG (34). Additionally, properdin treatment resulted in an increased CMC-CNTs (carboxy-methyl cellulose coated carbon nanotubes) uptake and significant induction of pro-inflammatory response by THP-1 cells (53).

IAV infection is significantly dependent on cellular factors for viral replication and infectivity. As a critical pro-inflammatory cytokine, TNF-α production triggers the activation of NF-κB, a major signalling transcription factor activated upon IAV infection, which initiates a strong type 1 IFN immune response (76, 77). Transcriptional profiling studies have reported that TNF receptor 1 knockout mice infected with the 1918 H1N1 IAV strain showed suppressed expression of genes involved in antiviral signalling pathways (78). Thus, signalling through TNF receptor-1 contributed significantly to the severity of the 1918 influenza pandemic (78); the systemic TNF-α levels were a crucial marker for the progression of the infection. NF-κB is regarded as a major regulator of innate immune surveillance against viral infection (76) due to its ability to trigger the expression of an array of cytokines and chemokines (79–81). IAV infection can trigger expression of IFN *via* NF-κB as viral titre was found to be significantly higher in cells treated with an anti-IFN-α/β receptor antibody, but not in dominant-negative IκBα expressing cells (76). In the current study, suppressed mRNA level of NF-κB was observed in properdin treated H1N1 challenged A549 cells at 6h, while upregulation was seen in the case of H3N2-challenged cells following properdin treatment. Furthermore, the NF-κB luciferase reporter assay also revealed a reduction in NF-κB expression levels in A549 cells challenged with H1N1 preincubated with properdin, while enhanced activity was detected in the case of H3N2 challenged cells.

Pseudotyped lentiviral particles expressing matched H1N1 and unmatched H3N2 glycoproteins were generated to study the effect of properdin treatment on IAV entry. The pseudotyped particles were generated using a HIV backbone that expresses firefly luciferase (pHIV-Luciferase), single packaging plasmid (psPAX2) that encodes the Gag, Pol, and Tat genes, and an envelope plasmid that encode their respective IAV glycoproteins. The HIV backbone used is “replication incompetent” as it contains an additional sequence deletion in the 3′ LTR that leads to viral “self-inactivation” after integration with the host DNA. This was selected as a safe alternative method to mimic the structure and surfaces of IAV, and to study the effect of properdin in viral entry. Properdin treatment was found to reduce luciferase reporter activity in MDCK cells transduced with matched H1N1 pseudotyped lentiviral particles. However, an enhanced luciferase reporter activity was observed in case of cells transduced with the unmatched H3N2 pseudotyped lentiviral particles, indicative of an entry inhibitory role of properdin against infectivity of H1N1 subtype, but not for H3N2.

To assess if properdin treatment inhibited the viral infection by binding to a cell-surface receptor or preventing viral endocytosis, a cell-binding assay was performed. It was found that properdin treatment caused a reduction in cell binding in A549 cells infected with the H1N1 subtype, while an enhanced cell binding was observed in the case of H3N2-infected cells when compared to untreated control (Cells+ virus only). This data seemed to suggest that properdin treatment was efficient in preventing viral particles from binding to their cell surface receptors in H1N1-infected A549 cells and thereby acting as an entry inhibitory against H1N1-subtype.

*In-silico* docking analysis revealed molecular interactions that may be involved in the properdin mediated selective inhibition of H1N1. Although the binding sites of the top docked poses of properdin with HA of H1N1 and H3N2 were proximal, there were few striking differences in the intermolecular interactions with residues of HA that are known to be important for virus infectivity. HA is a cylinder-shaped trimeric glycoprotein that is present in IAV viral membrane. Each HA trimer is made of 3 identical HA monomers. Each monomer consists of a HA precursor protein, a single HA0 polypeptide chain with HA1 and HA2 regions linked by 2 disulfide bridges (82, 83). The proteolytic activation of HA is a crucial determinant of viral infectivity (84, 85). This precursor form of HA, HA0, helps prevent premature and unwanted fusion activity and must be cleaved by host proteases to be infectious. The tropism and pathogenicity of the IAV strains are determined by the tissue-specific cellular proteases of the host that mediate the cleavage of HA0 into HA1 and HA2 (86). Properdin was found to interact with the cleavage site of HA of H1N1, but not of H3N2. The selective interaction of properdin with the HA cleavage site of H1N1 may explain the subtype dependent entry inhibitor activity observed between H1N1 and H3N2. It is also possible that this variation and differential binding capacity further affects downstream interactions such as IAV or properdin interaction with other cellular receptors or co-binding proteins that may affect viral entry or downstream signalling cascade.

In light of the current data establishing the specific interactions between properdin and IAV, we hope to examine future host response in the murine models of infection using wild type and properdin knockout mice (87, 88). Furthermore, it would also be worthwhile to study the level of properdin in the local microenvironment and in systemic circulation in human patients suffering from IAV infection and healthy volunteers.

In conclusion, this study indicated that the extent of immunomodulatory effects of properdin on host cells could differ depending on IAV subtypes. Entry inhibition is coupled with an anti-inflammatory response following properdin treatment in the case of H1N1, as evident from downregulation of IL-6, IL-12, TNF-α, IFN-α and NF-κB mRNA levels. However, with H3N2, properdin treatment upregulated these cytokines, highlighting the notion that properdin creates a pro-inflammatory milieu during IAV infection. As an HA or NA-based inhibitor against IAV infection, properdin treatment may be a viable therapeutic agent against the H1N1 subtype that may be administered as an inhalation formulation to control IAV infection.

## Data Availability Statement

The original contributions presented in the study are included in the article/**Supplementary Material**. Further inquiries can be directed to the corresponding authors.

## Ethics Statement

Human peripheral blood neutrophils were prepared using heparinized whole blood obtained from healthy donors, in accordance with King Faisal Specialist Hospital and Research Centre Research Advisory Council (IRP) regulations. The patients/participants provided their written informed consent to participate in this study.

## Author Contributions

PV, SM, FA-M, SS, and VM carried out crucial experiments. PV and VM conducted majority of the experiments. NB, SA, RR, BN, and RS provided/generated crucial reagents. SI-T, TM, and UK supervised different parts of the work. PV, VM, TM, and UK produced various drafts of the manuscript. All authors contributed to the article and approved the submitted version.

## Funding

SI-T acknowledges funding from the Department of Biotechnology (DBT), India [BT/PR40165/BTIS/137/12/2021]. SM acknowledges the Department of Science and Technology [SR/WOS-A/LS-38/2018] for a research fellowship. SA acknowledges funding by Researchers Supporting Project (RSP 2021/26), King Saud University, Riyadh, Saudi Arabia.

## Conflict of Interest

The authors declare that the research was conducted in the absence of any commercial or financial relationships that could be construed as a potential conflict of interest.

## Publisher’s Note

All claims expressed in this article are solely those of the authors and do not necessarily represent those of their affiliated organizations, or those of the publisher, the editors and the reviewers. Any product that may be evaluated in this article, or claim that may be made by its manufacturer, is not guaranteed or endorsed by the publisher.

## Supplementary Material

Supplementary Figure 1 Source blots demonstrating properdin **(A)** release from neutrophils during IAV infection. β-actin **(B)** is used as a loading control. 40µg total protein/well from the supernatants of freshly purified human peripheral blood neutrophils (Lane 1; Control), 24h H1N1 (0.1 PFU) challenged neutrophils (Lane 2; Treated), fMLP (1µM) treated neutrophils (Lane 3; positive control) and IL-6, (10 ng/ml; Sigma) (lane 4; positive control) were collected, concentrated, and subjected to SDS-PAGE separation in 1 x MOPS buffer (4-12%, NuPAGE gel). 10µg β-actin was added to each treated sample as a loading control prior to SDS-PAGE. Secreted properdin was detected by Western Blotting using anti-properdin (1: 2000 dilution), and actin was detected by rabbit anti-actin antibody (1:13000 dilution). ECL was performed using standard HRP-labelled secondary antibodies that were exposed for 2 seconds.

Supplementary Figure 2 Properdin treatment does not affect A549 cell viability. 3-(4,5-dimethylthiazol-2,5-diphenyltetrazolium bromide (MTT) assay was performed by seeding A549 cells (1 × 10^4^ cells/well) in a 96-well microtiter plate. The monolayers formed were treated for 24h with properdin (20 μg/ml), under anaerobic conditions at 37°C. Samples that were treated with a similar amount of properdin’s vehicle was used as control samples. The assays were performed following the manufacturer’s recommendations, and cell viability was measured by determining the absorbance at 570 nm. The background was subtracted from all data points. The data obtained were normalised with 100% cell viability being defined as the mean of the absorbance recorded from the control sample. The data were presented as the mean of the normalised triplicates ±. Significance was determined using the two-way ANOVA (n = 3). No significant difference in cell viability was observed between the samples treated with properdin.

Supplementary Figure 3 Properdin treatment does not affect cytokine mRNA expression in A549 cells. mRNA expression levels of selected cytokines and chemokines (TNF-α, IL-12, IL-6, RANTES, IFN-α and NF-κB) in A549 cells treated with only properdin (20µg/ml) for 2h (A) or 6h (B) were measured using qRT-PCR using the primers listed in . The data were normalised *via* 18S rRNA expression as an endogenous control. The relative expression (RQ) was calculated using the untreated sample (cells + properdin’s vehicle) as the calibrator. The RQ value was calculated using the formula: RQ = 2^−ΔΔCt^. Assays were conducted in triplicates, and error bars represent ± SEM. Significance was determined using the two-way ANOVA test (ns no significance) (n = 3).
