# Supplementary figures and images for: Human Properdin Released By Infiltrating Neutrophils Can Modulate Influenza A Virus Infection
Source: Front Immunol. 2021 Dec 9;12:747654. doi: 10.3389/fimmu.2021.747654 (PMC8695448; doi:10.3389/fimmu.2021.747654)

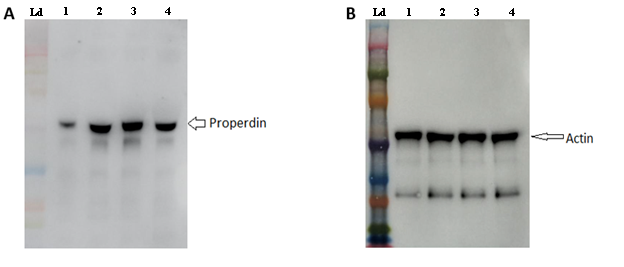

Supplement: Supplementary Figure 1 — Source blots demonstrating properdin (A) release from neutrophils during IAV infection. β-actin (B) is used as a loading control. 40µg total protein/well from the supernatants of freshly purified human peripheral blood neutrophils (Lane 1; Control), 24h H1N1 (0.1 PFU) challenged neutrophils (Lane 2; Treated), fMLP (1µM) treated neutrophils (Lane 3; positive control) and IL-6, (10 ng/ml; Sigma) (lane 4; positive control) were collected, concentrated, and subjected to SDS-PAGE separation in 1 x MOPS buffer (4-12%, NuPAGE gel). 10µg β-actin was added to each treated sample as a loading control prior to SDS-PAGE. Secreted properdin was detected by Western Blotting using anti-properdin (1: 2000 dilution), and actin was detected by rabbit anti-actin antibody (1:13000 dilution). ECL was performed using standard HRP-labelled secondary antibodies that were exposed for 2 seconds. [file Image_1.tif]

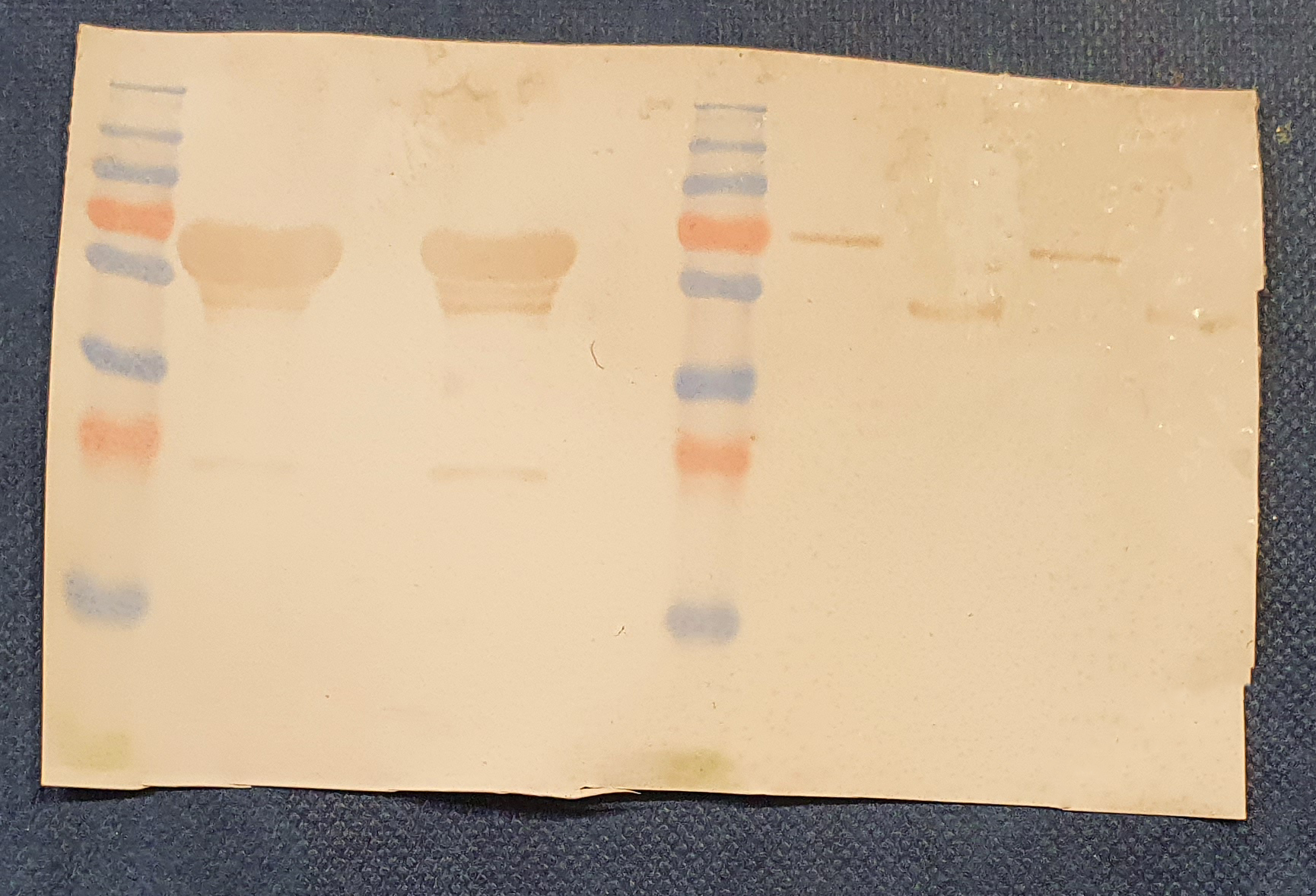

Supplement: Supplementary Figure 2 — Properdin treatment does not affect A549 cell viability. 3-(4,5-dimethylthiazol-2,5-diphenyltetrazolium bromide (MTT) assay was performed by seeding A549 cells (1 × 10^4^ cells/well) in a 96-well microtiter plate. The monolayers formed were treated for 24h with properdin (20 μg/ml), under anaerobic conditions at 37°C. Samples that were treated with a similar amount of properdin’s vehicle was used as control samples. The assays were performed following the manufacturer’s recommendations, and cell viability was measured by determining the absorbance at 570 nm. The background was subtracted from all data points. The data obtained were normalised with 100% cell viability being defined as the mean of the absorbance recorded from the control sample. The data were presented as the mean of the normalised triplicates ±. Significance was determined using the two-way ANOVA (n = 3). No significant difference in cell viability was observed between the samples treated with properdin. [file Image_2.tif]

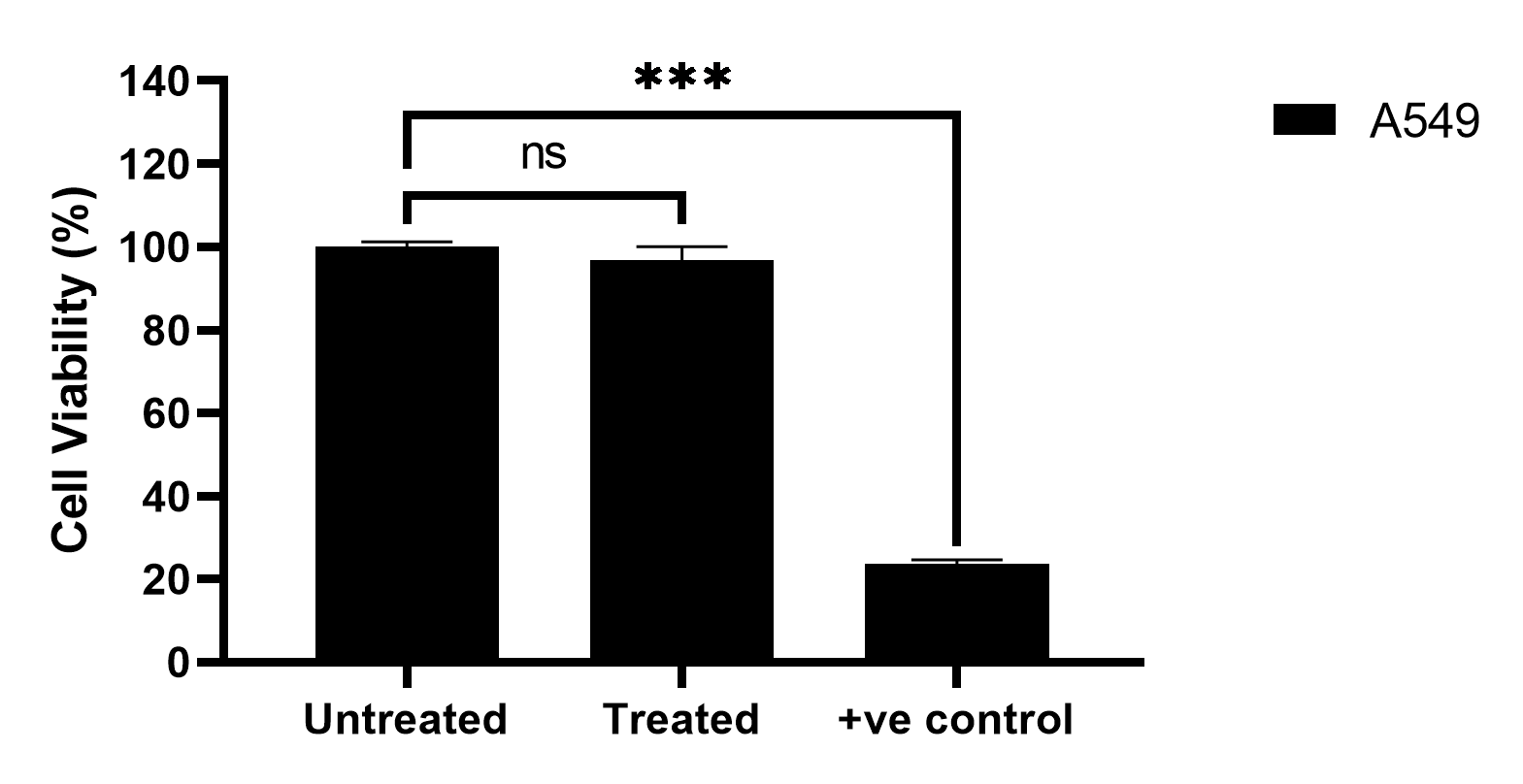

Supplement: Supplementary Figure 3 — Properdin treatment does not affect cytokine mRNA expression in A549 cells. mRNA expression levels of selected cytokines and chemokines (TNF-α, IL-12, IL-6, RANTES, IFN-α and NF-κB) in A549 cells treated with only properdin (20µg/ml) for 2h (A) or 6h (B) were measured using qRT-PCR using the primers listed in . The data were normalised via 18S rRNA expression as an endogenous control. The relative expression (RQ) was calculated using the untreated sample (cells + properdin’s vehicle) as the calibrator. The RQ value was calculated using the formula: RQ = 2−ΔΔCt. Assays were conducted in triplicates, and error bars represent ± SEM. Significance was determined using the two-way ANOVA test (ns no significance) (n = 3). [file Image_3.tif]

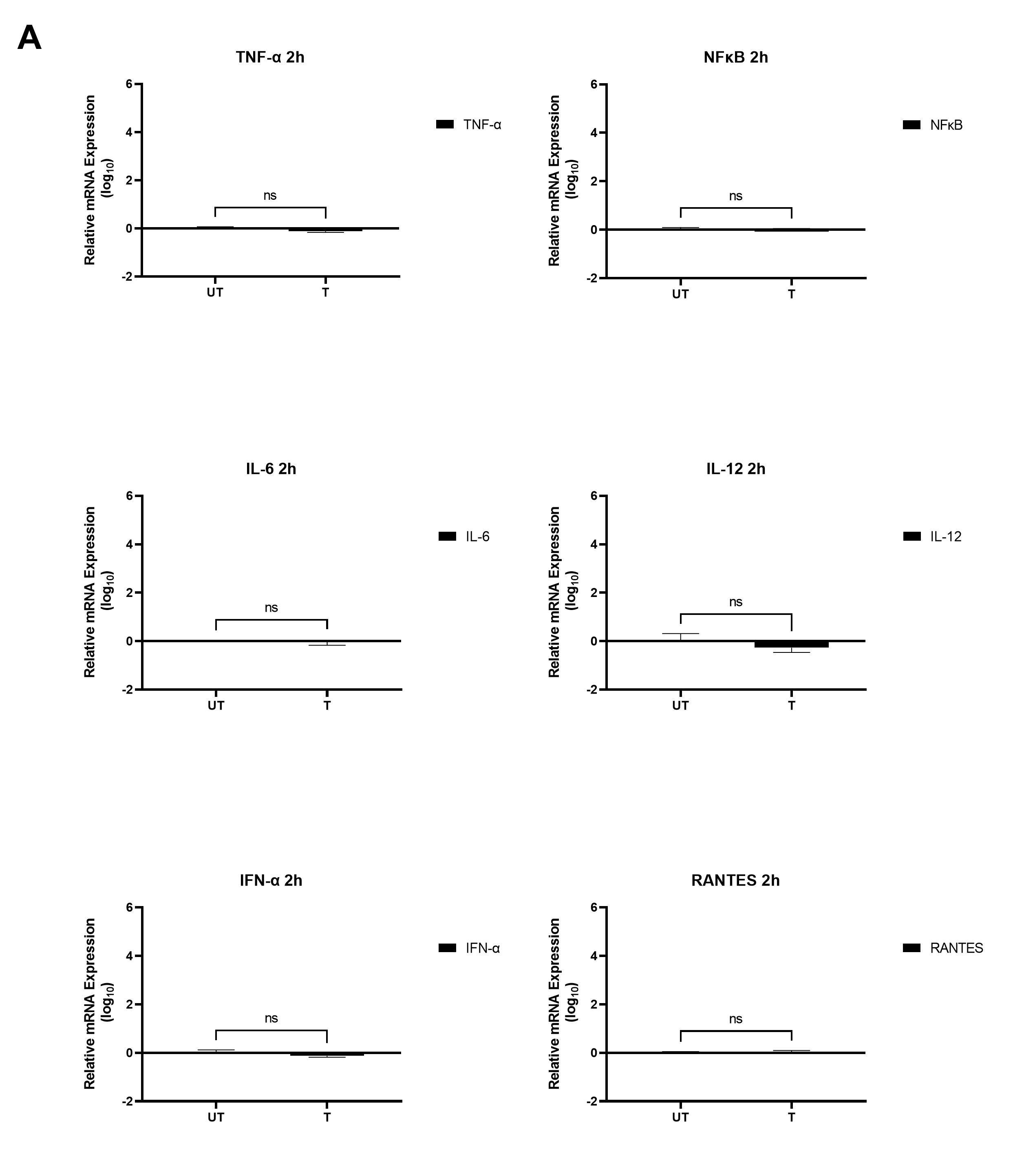

Supplement: Supplementary file 4 [file Image_4.tif]
